# Splendid Engravings of American Birds

**Published:** 1828-07

**Authors:** John James Audubon

**Affiliations:** No. 95, Great Russell Street, Bedford Square, London


16. *Splendid Engravings of American Birds.*—We have been lately favoured with the prospectus of a work on the birds of the United States, by Mr. J. J. Audubon, well known in these interior states, and for some time one of the artists of the Western Museum, in this city. When in that situation, we had frequent opportunities of inspecting his coloured crayon, drawings, of the birds of this country, and can bear testimony to their great beauty and fidelity. Mr. Audubon is now in London, where his publication is to be made. We deem the following extracts, from his prospectus not inappropriate, for a journal which circulates, extensively, in the region of country which has supplied the very originals to which he refers.

"To those who have not seen any portion of the author's splendid Collection of original Drawings, it may be proper to explain, that their superiority consists in every specimen being of the full size of life, portrayed with a degree of accuracy as to proportion and outline, the result of peculiar means discovered and employed by the author, and lately exhibited to a meeting of the Wernerian Society. Besides, in every instance where a difference of plumage exists between the sexes, both the male and the female Birds have been represented. The author has not contented himself with single profile views of the originals, but in very many instances he has grouped them, as it were, at their natural avocations, in all sorts of attitudes, either on branches of trees, or amidst plants or flowers. Some are seen pursuing with avidity their prey through the air, or searching diligently their food amongst the fragrant foliage; whilst others of an aquatic nature swim, wade, or glide over their allotted element. The insects, reptiles, or fishes, that form the food of the birds, have been introduced into the Drawings; and the nests of the birds have been frequently represented. The plants are all copied from nature, and the botanist, it is hoped, will look upon them with delight. The eggs of most of the species will appear in the course of publication.

"The great interest which has been excited by the exhibition of these Drawings, and the flattering praise which has been bestowed upon them in Edinburgh, Liverpool, and Manchester, have induced the author to publish Engravings of them, upon a scale of elegance never before attempted in this or any other country. He has been encouraged to commence such an arduous undertaking, at the suggestion of some of the most eminent naturalists both in America and Great Britain; and he is proud to acknowledge, that their patronage has been extended to him towards the encouragement of the work; and he trusts to their support and that of the public, enabling him to complete one of the most splendid publications which has ever appeared."

The work will consist of 300 figures, all as large as life; and coloured according to nature. When completed, it will cost about $560. We are happy to see, attached to the prospectus, a list of 120 subscribers, headed by George IV.

Gentlemen in the United States who wish to subscribe, may address themselves to *Mr. John James Audubon, No. 95, Great Russell Street, Bedford Square, London.*

